# Cholera Infantum

**Published:** 1873-08

**Authors:** Alex. S. Von Mansfelde

**Affiliations:** Chicago, Ill.


ARTICLE II.—*Cholera Infantum.* By ALEX. S. VON MANSFELDE, M.D., Chicago, Ill.

"Caloric not only expands living tissues, thereby diminishing vital affinity, but it also increases their susceptibility. It is chiefly by its power thus to diminish the tonicity or compactness of the tissues, while it increases their irritability, or susceptibility, that caloric, or a high temperature, becomes an efficient predisposing or existing cause of disease. Acting more directly on the cutaneous surface, and, both by continuity of structure and close physiological sympathy, on the whole internal mucous surface, also, a high atmospheric temperature renders these structures morbidly sensitive, while their expansion renders them more lax, and thereby puts them in the most favorable condition for sudden and rapid fluxes of fluids into and through them."—*Clinical Lectures of N. S. Davis, page* 151.

I am satisfied that the prime cause of summer complaint, which the learned Doctor calls "*high temperature*," is the identical trouble, and that teething, miasmata, etc., are minor accessories toward producing this so fatal disease of our children.

But I cannot agree with him in regard to the ultimate effect caloric has upon the vegetable or animal creation.—See *Medical and Surgical Reporter, Vol.* 25, *page* 9.

Is the expansion incident to the influence of heat the same all through the body? This is certainly not the case. It is an established fact, that solid bodies are better conductors of heat than fluids, and that heat only exerts its power through the interstices of atomic elements, which it has the power of enlarging. Now suppose that heat reaches the mucous apparati of the interior body, it will surely prove its influence first upon the cell walls of the mucous cell structures, and if it have any effect upon them, it will exert it before the cell contents shall perceive the slightest elevation of temperature.

Now this state of affairs is not admittable for certain organs only, for heat is not so partial, and yet the kidneys have suspended the normal action already before the fluxes from the intestinal canal take place. Does heat not affect the extensive mucous surface of that organ, or that of the lungs, the sniderian membrane and those of the liver?

It is always better to take for the explanation of our theory a single cell of a diseased or deranged organ, and perceive the changes produced in the same.

Take for an example a round cell of the rete mucosum of the skin and submit it to the influence of heat; *expansion* takes place, but of what? Certainly not of the whole cell, but simply of its cell walls; and what is the consequence? A flux of the cell contents, through the enlarged interstices, existing in its membranous cover, follows.

This egress of the cell fluid normally takes place by every *contraction* of the living cell, *absorbing* new materials by its *expansion* from the adjacent oxidized blood.

Now place in contact with the cell expanded by heat, after it has lost a part of its contents, oxidized blood, will it take up from this medium the part that shall establish the equilibrium? Or will it not take up particles of entirely different physical calibre to those absorbed by the natural process of imbibition and thus change at once the whole body from a physiological to a pathological condition? (perversion of structure). If so, does the condition of a patient suffering from summer complaint justify such deductions?

We can nowhere see a general change of the organs of the body in cholera infantum.

One more effect of heat upon organized bodies is the loss of their watery parts. We know that heat dries up vegetable and animal particles, extracting therefrom their liquid parts, which is the necessary solvent and producer of organic functions.

Please to take the example of the cell of the malpighian layer of the skin again, and observe the consequences of an abstraction of its fluid parts, and note the difference.

In the former case there would be a loss of fluid, which surely could readily be supplied; but here the material is wanting, the contractions and dilations go on, but in a lessened degree, until there is no more stimulus for dilating the *not contracted* but *collapsed* cells. This collapse of the general structure is hastened by the loss of tonicity, which is produced through the lessened work of those organs, whose secretions are the true food of the more vital parts of the body, especially the nervous system—we speak *mostly* of the *liver* and its *products*.

We wish to be understood, that expansion of the structures, is, the cell walls of the organic molecules, the cells, must be followed by hastened cell life, absorption and secretion of matter, until stagnation, the climax of such superwork, is reached; known as the pathological condition, inflammation.

Whereas the pathological index to cholera infantum is a general atonicity of the soft structures of the body, in no case to be allied with inflammation—it is the very opposite to this condition.

If our observation prove true, that is, that not excessive *expansion* by heat, but simply a lessened percentage of water, is the cause of summer complaint, we may justly be asked for a proof of association of cause and effect, and hasten to answer with a quotation from our article above referred to:

"The collapsed cells have no power to retain their fluid portion, and this oozes away through the pari passu prostrated excretory channels without restraint; and not only is this the case with the mucous tissues, but with the glandular structures and muscular fibres—they are all subject to the same law and consequences.

"These exudations and secretions, though governed by the same cause, will, under different circumstances, give rise to a different train of symptoms. In the stomach the acid secretion will produce upon the denuded surface an incontrolable effort at vomiting. The bowels are incapacitated to absorb any fluid; the brain, though at first painfully alive to the wants of the body, is lulled to rest by the same hand, till at last the exudate into the cranial cavity presses upon it, and soon the consequences are witnessed, nerve influence loses its power, and the last help vanishes.

"Will any one under such impressions venture to give a nerve sedative to control the action of the stomach, or prescribe chalk and magnesia usta controlling the acidity of the organic exudate, or an astringent to compel a lessened discharge from the bowels, driving the fluid so much the sooner into the cranial cavity, and hasten the advent of death, or give an anodyne to control the maniacal manifestations of anguish, knowing that only too soon the opposite state of affairs will take place?"—*Med. and Surg. Reporter*, *Vol.* 25, *No.* 1.

Referring to the all absorbing topic of treatment, we may be pardoned again if we quote from Dr. Davis' Clinical Lectures. On page 157 he says:

"Thus, in the first stage of those cases characterized by simple serous discharges, either by vomiting or purging, the indications are to allay the morbid irritability of the mucous membrane, and to increase the tone or contractility of the capillaries; to fulfill these requires the judicious combination of a tonic and anodyne."

The doctor asserts, on page 151, that *caloric* and its consequence, *expansion*, are the prime trouble; why does he not now, when he comes to the practical deductions of his pathology, keep up the chain of logic? Why not counteract *caloric* by its natural antagonist, *cold?*

Can this not be done very easily? How grateful it is to the body of the sufferer to be immersed into water somewhat colder than the body! Does he not seemingly regain his strength?

Or does the learned gentleman assert that the partaking of phloridzine, opium, sugar of lead, or calomel, lends wings to high atmospheric temperature?

We confess the good effect of the tonic with the stimulant to the liver and its accessories, but see no reason why the doctor should give them, excepting he answers the above question in the affirmative.

We assert that the cause of summer complaint is high temperature and its effect in lessening the percentage of the *watery ingredients* of the animal organism, thereby producing collapse of the cellular structures of the body, from which mechanically the watery element exudes.

Our therapeutic deductions are of the so-called allopathic style: "contraria contraribus curantur." We have a defect of water, and we supply it. Our cell structure works imperfectly, and is collapsed; we put it in a condition to resume healthy action by access to water, and encourage the trial by tonics and stimulants. We are aware of the lessened animal heat caused by the evaporation of the fluids of the body, and proven by the rapid consummation of all the fat of the system for that purpose; and we hasten to supply this material in the best manner possible. Our patient rapidly assumes the robes of health, and attests the correctness of our deductions.

Our saying and gainsaying may be somewhat incoherent for want of time for careful preparation, but our treatment of the disease stood the test of the last two summers, and was, to say the least, satisfactory to ourselves.

The defect of water was best replenished by baths of a medium temperature, followed by an inunction with a non-irritating oil; best is almond oil;—the object of the latter being a restriction of evaporation from the surface and the consequent loss of animal heat. It is also known to the profession, that in bathing, the body takes up a considerable amount of water.

In conjunction with this purely hygienic regimen, we give small doses of pepsine, subnitrate of bismuth and sugar, taking care to direct small doses repeatedly to be given, with a certain amount of water, milk-whey or milk, as the case may warrant, the prime object being the insurance of the administration of some fluid at stated intervals, which, without the medicine, in nine out of ten cases would be neglected, at the same time the latter aid the digestive apparati in their march to health.

Finally, we prescribe the following:

| | | |
|---|---|---|
| R. | Olei Morrhuæ, - - - - - - - | oz. ss. |
| | Emulsionis Gum. Trajacanthi, - - - - - | oz. jss. |
| | Quiniæ Sulphatis, - - - - - - - - | grs. iij. |
| | Chloroformi, - - - - - - }<br>Tincturæ Opii Simplicis, - - - - } | *aa*, gtts. x. |
| | Tincturæ Columbæ, - - - - - - - | dr. ij. |
| | Syrupi Ipecacuanhæ, - - - - - - - | dr. j. |
| | Syrupi Rhei Aromatici, - - - - - }<br>Syrupi Pruni Virginiensis, - - - } | *aa*, dr. ijss. |

A teaspoonful every hour or two to a child a year old.

The trajacanth emulsion is made by dissolving 2 drachms of gum trajacanth in 15 ozs. of water and 1 oz. of glycerine. A simple admixture of this emulsion with the other ingredients insures a perfect emulsion that will remain unchanged for a long time.

And what is the object of this medication?

The ol. morrhuæ, in combination with the gum, will cover the denuded surfaces over which it passes better than any other matter, and as the assimilation process is not altogether interfered with, some of the oil will be absorbed and aid in building up the lost animal heat.

The action of quinine is that of a substitute for an aliment lost by the inactivity of the liver, the province of which is either directly or indirectly to keep up the form of the cell structures, or their power of contraction and dilation.

The action of the columba and syr. rhei aromat. is that of excitors of liver function; but they are of benefit only when conditions are established that will favor renewed action—(*addition of fluid*)—otherwise they would be of no avail.

The ipecac, opium, and chloroform, guard the action of the other agents and keep the brain supplied with nourishment (*blood*), the anæmia of which is so dangerous to the patient.

The syrup of wild cherry is added to produce a better taste of the mixture.

In writing this article, we have no desire to simply criticise, for we know the value of the lectures above alluded to too well to be governed by so small a motive; but we wish to draw the attention of practitioners to this subject by daring to attack the lion, doing thus all we can for suffering childhood.

Since writing the above, our attention was drawn by Dr. F. Schaller, of this city, to an article in the "*Jahrbuch fur Kinderheilkunde*," No. 2, pages 176 and 177, written by Dr. Werthheimber, of Munich, subject, cholera infantum.

In this article the doctor advocates very strongly the administration of tea, good black China tea infusion. He foots upon the fact that the whole nervous system is relaxed, not only *per se*, but through the starvation process going on in the brain itself, anæmia of the same.

Tea, as well known, is a direct stimulant to the nerve centres and insures a renewed action of the same, and upon this action of the *drug* rests its good influence in cholera infantum.

But how does this agree with the anodyne principle of the lectures referred to?

